# Threshold Effect of C‐Reactive Protein‐Albumin‐Lymphocyte (CALLY) Index on Disease Activity in Rheumatoid Arthritis: Unveiling a Nonlinear Association

**DOI:** 10.1155/mi/9969741

**Published:** 2026-02-03

**Authors:** Lina Leng, Quanyi Tang, Ying Li, Jinfeng Zhang, Yaorong Han, Xiaoli Li

**Affiliations:** ^1^ Department of Rheumatology, Xingtai People’s Hospital, Xingtai, 054001, Hebei Province, China, xtrmyy.cn; ^2^ Department of Internal Medicine, Graduate School of Hebei Medical University, Shijiazhuang, 050017, Hebei Province, China, hebmu.edu.cn; ^3^ Department of Oncology, 82 Group Hospital of Chinese People’s Liberation Army, Baoding, 071000, Hebei Province, China

**Keywords:** biomarker, C-reactive protein-albumin-lymphocyte index, immunity, inflammation, nutritional, rheumatoid arthritis

## Abstract

The relationship between the C‐reactive protein (CRP)‐albumin‐lymphocyte (CALLY) index and disease activity of rheumatoid arthritis (RA) has not been explored at present. This study included 1058 RA patients and used a multiple linear regression model to evaluate the association between the CALLY index and 28 joint disease activity scores (DAS28) and further explored its potential nonlinear relationship using a two‐stage segmented linear regression model. Multivariate adjusted analysis showed that CALLY was significantly negatively correlated with DAS28‐erythrocyte sedimentation rate (ESR) (*β* = −0.119, 95% confidence interval [CI]: −0.145 to −0.093) and DAS28‐CRP (*β* = −0.201, 95% CI: −0.226 to −0.177) (both *p*  < 0.001), and there was a dose–response relationship (trend *p*  < 0.001). Segmented regression analysis revealed a significant nonlinear correlation between the two, with inflection points of 0.499 and 0.555, respectively. Below the inflection point, CALLY has a significant negative impact on disease activity (DAS28‐ESR: *β* = −2.102, 95% CI: −2.498 to −1.706); DAS28‐CRP: *β* = −2.311, 95% CI: −2.591 to −2.031); after exceeding the inflection point, this negative correlation effect significantly weakens but still maintains statistical significance. The results of this study indicate a significant nonlinear relationship between the CALLY index and the DAS28 score, especially at low CALLY levels. This study suggests the potential of CALLY as a novel composite biomarker reflecting the inflammatory status and disease severity of RA.

## 1. Introduction

Rheumatoid arthritis (RA) is a common chronic, systemic autoimmune disease characterized by persistent synovitis and joint destruction, ultimately leading to functional disability and decreased quality of life [1, 2]. RA has a significant global health burden, affecting ~0.5%–1% of the global population [3]. It is predicted that the global prevalence of RA will sharply increase, reaching 31.7 million by 2050 [4]. RA not only leads to functional impairment and decreased quality of life but also increases the risk of systemic complications, including cardiovascular events and interstitial lung disease [5]. At the same time, RA also causes a huge socioeconomic burden, which includes both the high direct medical costs of advanced biological therapies and the significant indirect costs incurred due to impaired work capacity [6, 7]. So, accurately assessing disease activity is crucial for guiding treatment decisions, achieving treatment goals (such as clinical remission or low disease activity), and improving long‐term prognosis.

At present, the disease activity score in 28 joints (DAS28) combined with erythrocyte sedimentation rate (ESR) or C‐reactive protein (CRP) is an indicator for evaluating RA disease activity in clinical practice and research [8]. However, the calculation of DAS28 is relatively complex, and ESR and CRP, as single inflammatory indicators, are susceptible to infection, anemia, and other factors, which limits their specificity and stability [9]. Therefore, the search for novel composite biomarkers that can more comprehensively and sensitively reflect the pathophysiological status of RA, including inflammation, nutrition, and immune disorders, has become an important research direction.

The CRP‐albumin‐lymphocyte (CALLY) index is a newly proposed indicator that integrates three key dimensions: inflammation (CRP), nutritional status (albumin), and immune status (lymphocytes) [10]. The CALLY index has been used for prognostic evaluation of various cancer patients, including those with gastric cancer, colorectal cancer with liver metastasis, and non‐small cell lung cancer [11–13]. In addition, the CALLY index is significantly negatively correlated with all‐cause mortality in elderly patients [14] and chronic obstructive pulmonary disease (COPD) patients [15]. However, the application and relevance of CALLY in RA have not been fully explored. Therefore, this study aims to elucidate the association between the CALLY index and RA disease activity, providing a new theoretical basis for precise risk assessment and clinical intervention of RA.

## 2. Methods

### 2.1. Study Population and Design

**Figure 1:**
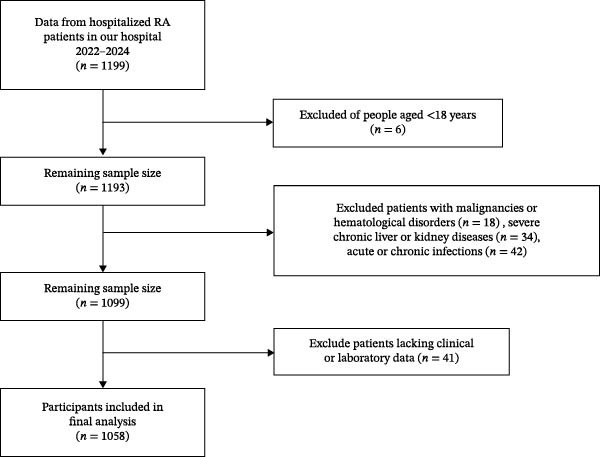
Flowchart of participant selection.

This study included RA patients admitted to Xingtai People’s Hospital between March 2022 and December 2024. In total 1199 patients diagnosed with RA were initially selected. The inclusion criteria are as follows: age ≥ 18 years old; meets the RA classification criteria proposed by the American College of Rheumatology (ACR)/European League Against Rheumatism (EULAR) in 2010 [16]. The exclusion criteria are as follows: age under 18 years old (*n* = 6); accompanied by a history of malignant tumors or a history of hematological disorders (*n* = 18); combined severe liver and kidney dysfunction (*n* = 34); acute or chronic infections (*n* = 42); and lack of clinical or laboratory data (*n* = 41). The screening process for research participants is shown in Figure 1. This study was approved by the Ethics Committee of Xingtai People’s Hospital and conducted in accordance with the ethical principles outlined in the Declaration of Helsinki. Written informed consent was obtained from all participants.

### 2.2. Data Collection and Variable Definition

Complete clinical and laboratory data were collected for all patients. These included (1) demographic data (sex and age); (2) clinical features (disease duration [MH] and body mass index [BMI]); (3) treatment status (use of glucocorticoids, use and number of conventional synthetic disease‐modifying antirheumatic drugs [csDMARDs: 0, 1, or ≥ 2], and use of biologic or targeted synthetic DMARDs [b/tsDMARDs]); and (4) laboratory parameters (white blood cell count [WBC], lymphocyte count [Lymph], neutrophil count [Neut], monocyte count [Mono], red blood cell count [RBC], hemoglobin [HGB], platelet count [PLT], albumin, globulin, ESR, and CRP). Disease activity was assessed by rheumatologists using the DAS28 based on ESR (DAS28‐ESR) and CRP (DAS28‐CRP). The CALLY index was calculated using the following formula:

CALLY=albumin (g/L)×lymphocyte count ×109/LCRP (mg/L).

### 2.3. Statistical Methods

Continuous variables are presented as mean ± standard deviation or median (interquartile range [IQR]) depending on their distribution; categorical variables are expressed as number (%). Group comparisons were performed using analysis of variance, the Kruskal–Wallis test, or the chi‐square test, as appropriate. To analyze the dose–response relationship between CALLY and disease activity, patients were stratified into tertiles based on CALLY values: T1 (lowest), T2, and T3 (highest). The association of each variable with DAS28‐ESR and DAS28‐CRP was first evaluated using simple linear regression to estimate *β* coefficients with 95% confidence intervals (CIs). To assess the independent association between CALLY and DAS28 scores, multivariable linear regression models were constructed with sequential adjustment: a crude model (unadjusted), Model I (adjusted for sex, age, disease duration, and BMI), and Model II (further adjusted for Neut, Mono, RBC, HGB, PLT, glucocorticoid use, b/tsDMARD use, and number of csDMARDs). A two‐piece linear regression model was used to identify a potential nonlinear relationship (inflection point) between CALLY and DAS28 scores. The statistical significance of the inflection point was tested using the likelihood ratio test, and regression coefficients were calculated separately on each side of the threshold. To evaluate the consistency of findings across disease stages, a subgroup analysis was performed by dividing patients into early RA (disease duration ≤ 24 months) and established RA (> 24 months) groups; all main analyses were repeated within each subgroup. All statistical analyses were performed using IBM SPSS Statistics (Version 25.0) and R software (Version 4.3.3) with relevant packages. A two‐sided *p*‐value < 0.05 was considered statistically significant.

## 3. Result

### 3.1. Baseline Characteristics of the Study Population

A total of 1058 RA patients were included in this study. The cohort comprised 226 males (21.40%) and 832 females (78.60%), with a median age of 59.00 years (IQR: 51.00–68.00). The median disease duration was 70.0 months (IQR: 12.00–132.00), and the BMI was 23.71 kg/m^2^ (IQR: 21.26–26.04). The distribution of the CALLY index was right‐skewed, with a median of 0.20 and an IQR of 0.10–0.70 (full range: 0.01–9.98). As shown in Table 1, baseline characteristics differed significantly across the CALLY tertile groups (except for glucocorticoid and biologic agent use). Compared to patients in the lowest CALLY tertile (T1), those in the highest tertile (T3) were more frequently female, younger, had a shorter disease duration, a higher BMI, and were more likely to be treated with csDMARDs—particularly with combination therapy (≥ 2 agents). More importantly, with the increase of CALLY levels, indicators reflecting inflammation (such as ESR, CRP, Neut, and platelets) significantly decreased (*p*  < 0.05), while nutritional and immune status indicators (such as Lymph, red blood cells, HGB, and albumin) significantly improved (*p*  < 0.05). Correspondingly, both DAS28 scores significantly decreased with increasing CALLY levels (DAS28‐ESR: T1 5.02 [4.57–5.41] vs. T3 3.91 ± 0.76; DAS28‐CRP: T1 4.40 [3.99–4.74] vs. T3 3.18 [2.66–3.53], *p* for trend < 0.001).

**Table 1:** General feature description according to tertiles of CALLY.

Characteristics	Total	Tertiles of CALLY			
		T1 (0.27–1.09)	T2 (1.09–1.30)	T3 (1.30–2.33)	*p*‐Value
*N*	1058	353	353	352	—
Sex (%)					< 0.001
Female	832 (78.60%)	250 (70.82%)	275 (77.90%)	307 (87.22%)	
Male	226 (21.40%)	103 (29.18%)	78 (22.10%)	45 (12.78%)	
Age (year)	59.00 (51.00–68.00)	63.00 (54.00–70.00)	59.00 (51.50–69.00)	55.00 (45.00–64.00)	< 0.001
MH (month)	70.00 (12.00–132.00)	78.00 (19.50–162.00)	68.00 (12.00–120.00)	60.00 (12.00–120.00)	0.018
BMI (kg/m^2^)	23.71 (21.26–26.04)	23.26 ± 3.57	24.05 ± 3.41	23.67 (21.49–26.51)	0.002
Glucocorticoids (%)					0.704
No	943 (89.10%)	311 (88.10%)	315 (89.24%)	317 (90.06%)	
Yes	115 (10.90%)	42 (11.90%)	38 (10.76%)	35 (9.94%)	
csDMARDs (%)					0.001
No	552 (52.20%)	201 (56.94%)	195 (55.24%)	156 (44.32%)	
Yes	506 (47.80%)	152 (43.06%)	158 (44.76%)	196 (55.68%)	
Type of csDMARDs (%)				—	0.009
0	552 (52.20%)	201 (56.94%)	195 (55.24%)	156 (44.32%)	
1	374 (35.30%)	110 (31.16%)	119 (33.71%)	145 (41.19%)	
≥2	132 (12.50%)	42 (11.90%)	39 (11.05%)	51 (14.49%)	
b/tsDMARDs (%)					0.335
No	985 (93.10%)	331 (93.77%)	332 (94.05%)	322 (91.48%)	
Yes	73 (6.90%)	22 (6.23%)	21 (5.95%)	30 (8.52%)	
ESR (mm/H)	61.00 (37.00–89.00)	90.00 (64.50–112.00)	66.00 (43.00–85.00)	35.00 (22.25–51.00)	< 0.001
CRP (mg/L)	29.94 (8.40–49.75)	63.02 (45.83–92.56)	30.76 ± 12.93	5.41 (2.43–8.63)	< 0.001
WBC (×10^9^/L)	6.11 (4.91–7.75)	6.32 (5.11–8.24)	6.54 (5.29–8.10)	5.64 (4.51–6.90)	< 0.001
Neut (×10^9^/L)	3.94 (2.88–5.28)	4.36 (3.37–5.98)	4.11 (3.11–5.38)	3.25 (2.49–4.47)	< 0.001
Lymph (×10^9^/L)	1.49 (1.16–1.89)	1.22 (0.96–1.51)	1.67 (1.32–2.06)	1.66 (1.32–2.03)	< 0.001
Mono (×10^9^/L)	0.44 (0.34–0.58)	0.47 (0.35–0.62)	0.45 (0.35–0.58)	0.41 (0.33–0.53)	< 0.001
RBC (×10^12^/L)	3.86 ± 0.50	3.70 ± 0.49	3.92 ± 0.48	3.97 ± 0.49	< 0.001
HGB (g/L)	110.00 (99.00–122.00)	102.92 ± 16.26	113.00 (103.00–124.00)	115.37 ± 15.89	< 0.001
PLT (×10^9^/L)	278.00 (223.00–344.00)	302.00 (244.00–379.00)	294.59 ± 92.73	257.00 (212.25–301.75)	< 0.001
Albumin (g/L)	36.65 ± 5.02	33.60 (30.75–36.75)	37.17 ± 4.05	39.14 ± 4.05	< 0.001
Globulin (g/L)	32.30 (28.80–36.20)	34.30 (30.50–38.20)	33.16 ± 5.54	30.25 (27.20–33.60)	< 0.001
DAS28‐ESR	4.57 (3.96–5.08)	5.02 (4.57–5.41)	4.63 (4.18–5.04)	3.91 ± 0.76	< 0.001
DAS28‐CRP	3.84 (3.28–4.33)	4.40 (3.99–4.74)	3.96 (3.59–4.27)	3.18 (2.66–3.53)	< 0.001

*Note:* CALLY, C‐reactive protein‐albumin‐lymphocyte index; CRP high‐sensitivity, C‐reactive protein; csDMARDs, disease‐modifying antirheumatic drugs; DAS28, 28‐joint disease activity score; Lymph, lymphocyte count; Mono, monocyte count; Neut, neutrophil count; WBC, white blood cell count.

Abbreviations: b/tsDMARDs, biologic or targeted synthetic DMARDs; BMI, body mass index; ESR, erythrocyte sedimentation rate; HGB, hemoglobin; MH, medical history; PLT, platelet; RBC, red blood cell.

In addition, to explore the clinical significance of extremely high CALLY values, we defined patients with CALLY levels above the 90th percentile (> 2.16) as “highest CALLY decile” (*n* = 105) and compared them with all other patients (*n* = 953). As shown in Table S1, patients in the highest CALLY decile subgroup had better disease activity: their DAS28‐ESR and DAS28‐CRP scores were significantly reduced, and the proportion of patients achieving clinical remission was higher. Better indicator components are as follows: their serum albumin levels are higher, CRP levels are extremely low, and Lymphs are higher.

The detailed profile of antirheumatic drug use at baseline is summarized in Table S2. In brief, 35.30% of patients were receiving at least one csDMARD, with methotrexate being the most common (19.19%). The overall usage of b/tsDMARDs was 6.9%, comprising TNF inhibitors (3.78%), tocilizumab (0.38%), and tofacitinib (2.74%).

### 3.2. Univariate Analysis of Factors Affecting RA Disease Activity

The results of univariate linear regression analysis (Table 2) show that multiple variables are significantly correlated with DAS28 scores. Older age, higher ESR, CRP, WBC, neutrophils, monocytes, platelets, and globulin levels are risk factors for increased disease activity (*β* > 0, *p*  < 0.05). The use of csDMARDs/biologics, higher levels of red blood cells, HGB, and albumin were protective factors (*β* < 0, *p*  < 0.05). Of particular note, the CALLY index showed a significant negative correlation with both DAS28 scores (DAS28‐ESR: *β* = −0.220, 95% CI: −0.249 to −0.191; DAS28‐CRP: *β* = −0.277, 95% CI: −0.302 to −0.252, both *p*  < 0.001).

**Table 2:** The results of univariate analysis.

Characteristics	Statistics	DAS28‐ESR *β* (95% CI)	P_ERS_ value	DAS28‐CRP *β* (95% CI)	P_CRP_ value
Sex *N* (%)					
Female	832 (78.60%)	Ref	—	Ref	—
Male	226 (21.40%)	−0.107 (−0.224, 0.011)	0.075	−0.288 (−0.400, −0.177)	0.000
Age (year)	59.00 (51.00–68.00)	0.012 (0.008, 0.016)	0.000	0.011 (0.008, 0.015)	0.000
MH (month)	70.00 (12.00–132.00)	0.000 (0.000, 0.001)	0.665	0.000 (0.000, 0.001)	0.342
BMI (kg m^2^)	23.71 (21.26–26.04)	−0.014 (−0.027, 0.000)	0.045	−0.011 (−0.024, 0.002)	0.091
Glucocorticoids *N* (%)					
No	943 (89.10%)	Ref	—	Ref	—
Yes	115 (10.90%)	−0.108 (−0.263, 0.047)	0.171	−0.093 (−0.241, 0.056)	0.220
csDMARDs *N* (%)					
No	552 (52.20%)	Ref	—	Ref	—
Yes	506 (47.80%)	−0.289 (−0.384, −0.194)	0.000	−0.308 (−0.398, −0.217)	0.000
Type of csDMARDs					
0	552 (52.20%)	Ref	—	Ref	—
1	374 (35.30%)	−0.151 (−0.251, −0.050)	0.003	−0.195 (−0.291, −0.099)	0.000
≥ 2	132 (12.50%)	−0.345 (−0.490, −0.201)	0.000	−0.294 (−0.433, −0.156)	0.000
b/tsDMARDs					
No	985 (93.10%)	Ref	—	Ref	—
Yes	73 (6.90%)	−0.326 (−0.516, −0.137)	0.001	−0.299 (−0.480, −0.117)	0.001
ESR (mm/H)	61.00 (37.00–89.00)	0.018 (0.017, 0.019)	0.000	0.014 (0.013, 0.015)	0.000
CRP (mg/L)	29.94 (8.40–49.75)	0.009 (0.008, 0.010)	0.000	0.012 (0.011, 0.012)	0.000
WBC (×10^9^/L)	6.11 (4.91–7.75)	0.079 (0.059, 0.100)	0.000	0.107 (0.087, 0.126)	0.000
Neut (×10^9^/L)	3.94 (2.88–5.28)	0.107 (0.083, 0.131)	0.000	0.140 (0.118, 0.162)	0.000
Lymph (×10^9^/L)	1.49 (1.16–1.89)	−0.019 (−0.099, 0.062)	0.652	−0.019 (−0.096, 0.058)	0.630
Mono (×10^9^/L)	0.44 (0.34–0.58)	0.428 (0.0174, 0.682)	0.001	0.674 (0.433, 0.915)	0.000
RBC (×10^12^/L)	3.86 ± 0.50	−0.364 (−0.458, −0.270)	0.000	−0.183 (−0.275, −0.191)	0.000
HGB (g/L)	110.00 (99.00–122.00)	−0.013 (−0.016, −0.011)	0.000	−0.009 (−0.011, −0.006)	0.000
PLT (×10^9^/L)	278.00 (223.00–344.00)	0.002 (0.002, 0.003)	0.000	0.002 (0.002, 0.003)	0.000
Albumin (g/L)	36.65 ± 5.02	−0.056 (−0.065, −0.047)	0.000	−0.057 (−0.066, 0.049)	0.000
Globulin (g/L)	32.30 (28.80–36.20)	0.068 (0.061, 0.076)	0.000	0.052 (0.044, 0.059)	0.000
CALLY	0.20 (0.10–0.70)	−0.220 (−0.249, −0.191)	0.000	−0.277 (−0.302, −0.252)	0.000

*Note:* CALLY, C‐reactive protein‐albumin‐lymphocyte index; CRP high‐sensitivity, C‐reactive protein; csDMARDs, disease‐modifying antirheumatic drugs; DAS28, 28‐joint disease activity score; Lymph, lymphocyte count; Mono, monocyte count; Neut, neutrophil count; WBC, white blood cell count.

Abbreviations: b/tsDMARDs, biologic or targeted synthetic DMARDs; BMI, body mass index; ESR, erythrocyte sedimentation rate; HGB, hemoglobin; MH, medical history; PLT, platelet; RBC, red blood cell.

### 3.3. Multivariate Linear Regression Analysis of CALLY and RA Disease Activity

After adjusting for multiple potential confounding factors, multivariate linear regression analysis (Table 3) showed that in fully adjusted Model II, for every unit increase in CALLY, the DAS28‐ESR score significantly decreased by 0.119 points (*β* = −0.119, 95% CI: −0.145 to −0.093), and the DAS28‐CRP score significantly decreased by 0.201 points (*β* = −0.201, 95% CI: −0.226 to −0.177). Referring to the lowest tertile array of CALLY (T1), the DAS28 scores of the middle group (T2) and high tertile group (T3) showed a dose‐dependent decrease (trend *p*‐value < 0.001).

**Table 3:** Multivariate linear regression results of association between CALLY and DAS28.

Exposure	Crude model *β* (95% CI)	Adjust I *β* (95% CI)	Adjust II *β* (95% CI)
DAS28‐ESR			
CALLY	−0.220 (−0.249, −0.191)	−0.211 (−0.240, −0.182)	−0.119 (−0.145, −0.093)
CALLY (tertiles)			
T1	Ref	Ref	Ref
T2	−0.371 (−0.471, −0.271)	−0.371 (−0.472, −0.271)	−0.179 (−0.271, −0.087)
T3	−1.033 (−1.133, −0.933)	−1.022 (−1.125, −0.918)	−0.606 (−0.709, −0.503)
* p* for trend	0.000	0.000	0.000
DAS28‐CRP			
CALLY	−0.277 (−0.302, −0.252)	−0.266 (−0.291, −0.240)	−0.201 (−0.226, −0.177)
CALLY (tertiles)			
T1	Ref	Ref	Ref
T2	−0.439 (−0.522, −0.357)	−0.438 (−0.521, −0.355)	−0.344 (−0.425, −0.262)
T3	−1.267 (−1.350, −1.185)	−1.252 (−1.338, −1.167)	−1.023 (−1.114, −0.931)
* p* for trend	0.000	0.000	0.000

*Note:* Crude model adjusted for: none; Adjust I model adjusted for: sex, age, medical history, and BMI; Adjust II model adjusted for: sex, age, medical history, BMI, neutrophil count, monocyte count, red blood cell, hemoglobin, platelet, glucocorticoids, b/tsDMARDs, and type of csDMARDs. CALLY, C‐reactive protein‐albumin‐lymphocyte index; CRP high‐sensitivity, C‐reactive protein; csDMARDs, disease‐modifying antirheumatic drugs; DAS28, 28‐joint disease activity score.

Abbreviations: b/tsDMARDs, biologic or targeted synthetic DMARDs; BMI, body mass index; ESR, erythrocyte sedimentation rate.

### 3.4. Nonlinear Relationship Between CALLY and RA Disease Activity

Given that both univariate and multivariate analyses indicate significant associations, we further employed a two‐stage segmented linear regression model to explore their intrinsic relationships. The results (Table 4) revealed a strong nonlinear (inflection point) effect: the inflection point of the relationship between CALLY and DAS28‐ESR was 0.499 (Figure 2a), and the inflection point of the relationship with DAS28‐CRP was 0.555 (Figure 2b). On the left side of the inflection point (when CALLY levels are low), there is a steep negative correlation between CALLY and DAS28 scores (DAS28‐ESR: *β* = −2.102, 95% CI: −2.498 to −1.706; DAS28‐CRP: *β* = −2.311, 95% CI: −2.591 to −2.031). When the CALLY level exceeds the inflection point, its negative correlation with the DAS28 score significantly weakens but still maintains statistical significance (DAS28‐ESR: *β* = −0.083, 95% CI: −0.116 to −0.049; DAS28‐CRP: *β* = −0.103, 95% CI: −0.130 to −0.075). The likelihood ratio test results (*p*  < 0.001) indicate that the piecewise linear model can better fit the data than the simple linear model, confirming the authenticity of this nonlinear relationship.

Figure 2(a) A nonlinear relationship of CALLY with DAS28‐ESR. The model was adjusted for sex, age, medical history, BMI, neutrophil count, monocyte count, red blood cell, hemoglobin, platelet, glucocorticoids, b/tsDMARDs, and type of csDMARDs. (b) A nonlinear relationship of CALLY with DAS28‐CRP. The model was adjusted for sex, age; medical history, BMI, neutrophil count, monocyte count, red blood cell, hemoglobin, platelet, glucocorticoids, b/tsDMARDs, and type of csDMARDs. BMI, body mass index; CALLY, C‐reactive protein‐albumin‐lymphocyte index; CRP, high‐sensitivity C‐reactive protein; csDMARDs, disease‐modifying antirheumatic drugs; b/tsDMARDs, biologic or targeted synthetic DMARDs; DAS28, 28‐joint disease activity score; ESR, erythrocyte sedimentation rate.

**Figure:**
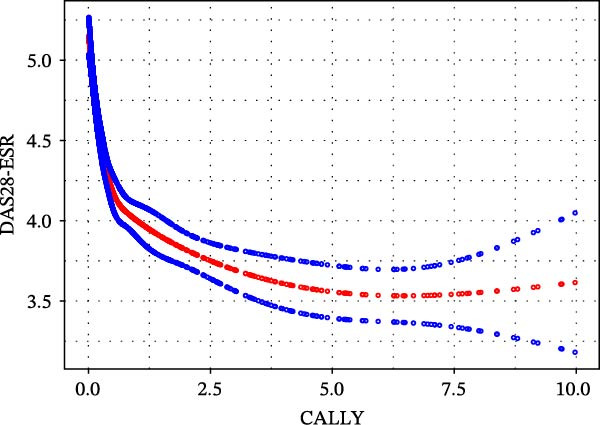
(a)

**Figure:**
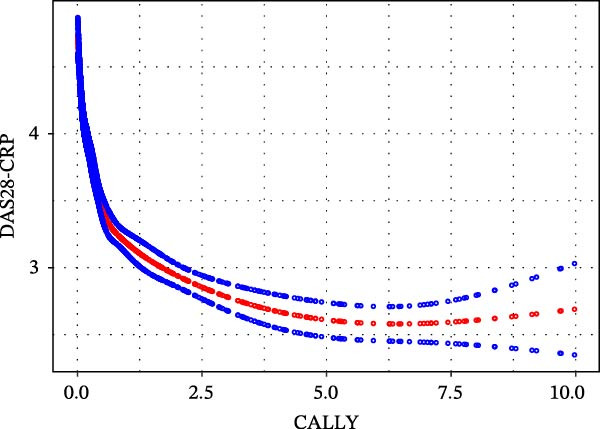
(b)

**Table 4:** The result of two‐piecewise linear regression model of CALLY with DAS28‐ESR and DAS28‐CRP.

Model and parameters	DAS28‐ESR *β* (95% CI)	DAS28‐CRP *β* (95% CI)
Fitting model by standard linear regression	4.472 (4.432, 4.511)	3.769 (3.738–3.800)
Fitting model by two‐piecewise linear regression		
Inflection points of CALLY	0.499	0.555
< Inflection point	−2.102 (−2.498, −1.706)	−2.311 (−2.591, −2.031)
> Inflection point	−0.083 (−0.116, −0.049)	−0.103 (−0.130, −0.075)
*p* for log likelihood ratio test	0.000	0.000

*Note:* The models were adjusted for sex, age, medical history, BMI, neutrophil count, monocyte count, red blood cell, hemoglobin, platelet, glucocorticoids, b/tsDMARDs, and type of csDMARDs.CALLY, C‐reactive protein‐albumin‐lymphocyte index; CRP, high‐sensitivity C‐reactive protein; csDMARDs, disease‐modifying antirheumatic drugs; DAS28, 28‐joint disease activity score.

Abbreviations: b/tsDMARDs, biologic or targeted synthetic DMARDs; BMI, body mass index; ESR, erythrocyte sedimentation rate.

### 3.5. Subgroup Analysis Stratified by Disease Course

To verify the universality of the association between CALLY and disease activity in different disease stages, we conducted subgroup analysis (baseline characteristics are shown in Table S3). The initial univariate analyses (Table S4) revealed that CALLY was significantly and negatively associated with both DAS28‐ESR and DAS28‐CRP in both early and established RA groups. In both the early RA group (*n* = 345) and the established RA group (*n* = 713), CALLY was significantly negatively correlated with DAS28‐ESR and DAS28‐CRP. After adjusting for multiple factors (Adjust II model), for every one unit increase in CALLY, DAS28‐ESR decreased by 0.141 points in the early RA group and 0.180 points in the established RA group. For every one unit increase in CALLY, DAS28‐CRP decreased by 0.212 points in the early RA group and 0.235 points in the established RA group (Table S5). CALLY tertile analysis and nonlinear segmented regression further confirm that the pattern and intensity of this association are highly consistent between the two groups: there is a significant dose–response trend (*p* for trend < 0.001) and similar inflection points (early RA group 0.407 and established RA group 0.551), with a steep slope of strong association below the inflection point (Table S6).

## 4. Discussion

To our knowledge, this study is the first large‐scale investigation into the relationship between the CALLY index and disease activity in RA. This study suggests that at baseline, a high CALLY index is significantly associated with more benign clinical features and lower disease activity. After adjusting for multiple potential confounding factors, CALLY remains a strong independent negative correlate of DAS28 score and exhibits a clear dose–response relationship. In addition, there is a significant nonlinear relationship between CALLY and DAS28 scores, rather than a simple linear correlation. This robust nonlinear model has been consistently validated in both DAS28‐ESR and DAS28‐CRP.

The pathogenesis of RA involves a complex interplay of genetic, epigenetic, environmental, metabolic, immune, and microbial factors [17, 18]. Its pathological process not only involves chronic inflammation but also often accompanies “inflammatory malnutrition” [19] and immune cell dysfunction [20]. As our understanding of the complex pathological mechanisms of RA deepens, a comprehensive assessment of inflammation, nutritional status, and immune function has become an important tool for optimizing individual outcomes and guiding treatment decisions. CRP is a widely recognized acute phase reactant synthesized by the liver in response to systemic inflammation and infection. It plays a crucial role in host defense mechanisms by activating the complement pathway and promoting phagocytosis [21]. Usually, CRP is routinely evaluated as a biomarker of systemic inflammation in RA [22], and increasing preclinical evidence suggests that CRP may play a direct role in bone destruction in RA [23]. Human serum albumin is the most abundant protein in plasma, playing a crucial role in maintaining osmotic pressure, regulating vascular permeability, and modulating inflammatory responses [24]. The concentration of serum albumin is related to the severity of malnutrition [25]. Research has shown that a decrease in albumin is associated with poorer pathological condition and physical function in RA, which coincides with adverse disease progression [26]. Meanwhile, the inflammatory process can exacerbate hypoalbuminemia through mechanisms such as inducing capillary leakage [27]. Lymphocytes are the smallest white blood cells and play an irreplaceable role in the body’s immune response [28]. The decrease in lymphocytes is the result of the continuous migration and infiltration of peripheral lymphocytes in the inflamed synovium of RA, and the upregulation of early apoptosis markers in peripheral blood lymphocytes may initiate a cascade reaction of apoptosis, thereby increasing lymphocyte apoptosis in RA patients [29]. In addition, diverse T cell subpopulations can recruit monocytes/macrophages, promote osteoblast differentiation, and produce inflammatory cytokines [30]. At the same time, B lymphocytes can produce immune complexes formed by autoantibodies such as rheumatoid factor and anti‐citrullinated protein antibodies to activate the complement pathway and cause the production of C5a and membrane attack complexes, thereby damaging joints [31]. By combining the CALLY index with representative indicators such as inflammation (CRP), nutrition (serum albumin), and immunity (lymphocytes), the independent associative value and interaction of these three indicators can be fully utilized. Compared to using CRP or ESR alone, CALLY can provide richer pathophysiological information, which explains why it exhibits such strong independent association in our multivariate model.

The observed right‐skewed distribution of CALLY is itself informative. Patients with very high CALLY values (highest decile, > 2.16) constituted a “deep remission” subgroup characterized by low inflammation, good nutritional status, and robust Lymphs. This distribution reflects the real‐world proportion of patients achieving this optimal state. Moreover, the observed effect size was clinically meaningful: the difference in DAS28‐ESR between the extreme CALLY tertiles was ~0.6, meeting the threshold for a minimal clinically important difference.

The CALLY index, as an emerging composite biomarker, has far less application in rheumatology than in oncology. However, preliminary evidence suggests its importance in RA. A recent study revealed a nonlinear L‐shaped association between CALLY and all‐cause mortality in RA patients, with a threshold of 12.79 [32]. Correspondingly, this study has found for the first time that there is a significant nonlinear “threshold effect” between CALLY and RA disease activity (DAS28). Although the outcomes of the two studies were different (long‐term mortality rate vs. current disease activity), they collectively pointed to a core feature: the clear critical level of protective effect of CALLY on RA patients. This inflection point delineates two clinical zones: a high‐risk critical zone (CALLY < 0.5), where small decreases in CALLY are associated with sharp rises in disease activity, marking a state of concurrent inflammation, malnutrition, and immune dysregulation, and a platform stability zone (CALLY > 0.5), where the association with disease activity plateaus. This suggests that maintaining CALLY above this threshold may be associated with a more stable disease state, though interventional studies are required to determine if modulating CALLY can be a valid treatment target. Subgroup analysis confirmed that the strong negative correlation and nonlinear threshold effect were consistent in both early (≤ 24 months) and established RA (> 24 months), enhancing the generalizability and robustness of our findings.

Previous studies have mostly focused on the association between a single indicator or other composite indicators, such as neutrophil‐to‐lymphocyte ratio (NLR), platelet‐to‐lymphocyte ratio (PLR), systemic immune‐inflammation index (SII), and RA. Our research introduces the new indicator CALLY into the field of RA and reveals for the first time its complex nonlinear relationship with disease activity, reminiscent of threshold effects observed with some prognostic biomarkers in oncology [33, 34]. Our results emphasize that in future research, simply adopting linear assumptions may mask or underestimate the true strength of the association between biomarkers and outcomes, especially at extreme values.

The advantages of this study include a large sample size, detailed clinical and laboratory data, rigorous multivariate adjustment, and innovative statistical methods. However, several limitations still need to be acknowledged. First, the cross‐sectional design precludes causal inference; we cannot determine whether low CALLY drives, results from, or bidirectionally relates to high disease activity. Second, the data come from a single center and may have selection bias, and external validity needs to be validated by a multicenter prospective cohort. Third, detailed data on drug dosage, duration, and treatment sequence were unavailable, restricting our ability to fully adjust for treatment effects. Fourth, the relatively low rate of biologic/targeted drug use may reflect local practice patterns, potentially limiting applicability to populations, where such therapies are standard. Finally, although we adjusted for multiple confounding factors, the possibility of residual confounding (such as diet, physical activity, and specific medication dosage) still exists. In future, prospective cohort studies are needed to validate the predictive ability of CALLY for clinical remission, radiological progression, and treatment response.

## 5. Conclusion

In summary, this study reveals a significant, independent, and nonlinear association between the CALLY index and disease activity in RA. The significant nonlinear inflection point suggests its potential utility for identifying distinct disease activity states. Future prospective studies are warranted to determine whether CALLY can serve as a reliable tool for risk stratification and to explore its potential role in guiding management strategies.

## Author Contributions

**Lina Leng**: conceptualization, data curation, formal analysis, investigation, methodology, resources, software, supervision, validation, visualization, writing – original draft, writing – review and editing. **Quanyi Tang**: conceptualization, data curation, investigation, methodology, software, visualization, writing – original draft. **Ying Li**: conceptualization, investigation, methodology, software, visualization, writing – original draft. **Jinfeng Zhang**: conceptualization, investigation, methodology, software, visualization. **Yaorong Han**: conceptualization, investigation, methodology, software, visualization. **Xiaoli Li**: conceptualization, funding acquisition, methodology, project administration, resources, supervision, visualization, writing – review and editing.

## Funding

This study was supported by Key R&D Projects in Xingtai City (Grant 2025ZC074).

## Ethics Statement

This study received approval from the Research Ethics Committee of Xingtai People’s Hospital (Approval Number: 2025 [031]). The studies were conducted in accordance with the local legislation and institutional requirements. The participants provided their written informed consent to participate in this study.

## Conflicts of Interest

The authors declare no conflicts of interest.

## Supporting Information

Additional supporting information can be found online in the Supporting Information section.

## Supporting information


**Supporting Information** Table S1: Comparison of clinical characteristics between patients in the highest decile of CALLY and all other patients. Table S2: Detailed use of antirheumatic drugs in the study cohort (*N* = 1,058). Table S3: Baseline characteristics by disease duration group. Table S4: Univariate analysis of factors associated with DAS28 by disease duration. Table S5: Multivariate linear regression by disease duration group. Table S6: Two‐Piecewise linear regression by disease duration.

## Data Availability

The data that support the findings of this study are available from the corresponding author upon reasonable request.
