# Supplementary material for: Threshold Effect of C‐Reactive Protein‐Albumin‐Lymphocyte (CALLY) Index on Disease Activity in Rheumatoid Arthritis: Unveiling a Nonlinear Association
Source: Mediators Inflamm. 2026 Feb 3;2026:9969741. doi: 10.1155/mi/9969741 (PMC12866333; doi:10.1155/mi/9969741)
Supplement: Supplementary file 1 — Supporting Information Table S1: Comparison of clinical characteristics between patients in the highest decile of CALLY and all other patients. Table S2: Detailed use of antirheumatic drugs in the study cohort (N = 1,058). Table S3: Baseline characteristics by disease duration group. Table S4: Univariate analysis of factors associated with DAS28 by disease duration. Table S5: Multivariate linear regression by disease duration group. Table S6: Two‐Piecewise linear regression by disease duration. [file MI-2026-9969741-s001.docx]

**Supplementary Table S1.** Comparison of Clinical Characteristics between Patients in the Highest Decile of CALLY and All Other Patients.

| Characteristics | Total | Highest CALLY Decile  (CALLY > 2.16) | All Other Patients  (CALLY ≤ 2.16) | *P* value |
| --- | --- | --- | --- | --- |
| N | 1058 | 105 | 953 |  |
| Sex, % | | | | 0.009 |
| Female | 832 (78.60%) | 93 (88.57%) | 214 (22.46%) |  |
| Male | 226 (21.40%) | 12 (11.43%) | 739 (77.54%) |  |
| Age (year) | 59.00 (51.00-68.00) | 54.94±13.74 | 59.00 (51.00-68.00) | 0.015 |
| MH (month) | 70.00 (12.00-132.00) | 48.00 (9.00-120.00) | 72.00 (13.50-137.50) | 0.148 |
| BMI (kg/m^2^) | 23.71 (21.26-26.04) | 23.71±3.10 | 23.74 (21.26-26.04) | 0.748 |
| Glucocorticoids,% | | | | 0.641 |
| No | 943 (89.10%) | 95 (90.48%) | 848 (88.98%) |  |
| Yes | 115 (10.90%) | 10 (9.52%) | 105 (11.02%) |  |
| csDMARDs, % | | | | 0.802 |
| No | 552 (52.20%) | 56 (53.33%) | 496 (52.05%) |  |
| Yes | 506 (47.80%) | 49 (46.67%) | 457 (47.95%) |  |
| Type of csDMARDs,% | | | | 0.966 |
| 0 | 552 (52.20%) | 56 (53.33%) | 496 (52.05%) |  |
| 1 | 374 (35.30%) | 36 (34.29%) | 338 (35.47%) |  |
| ≥2 | 132 (12.50%) | 13 (12.38%) | 119 (12.48%) |  |
| b/tsDMARDs,% | | | | 0.921 |
| No | 985 (93.10%) | 98 (93.33%) | 887 (93.07%) |  |
| Yes | 73 (6.90%) | 7 (6.67%) | 66 (6.93%) |  |
| ESR (mm/H) | 61.00 (37.00-89.00) | 28.00 (15.00, 41.50) | 65.00 (41.00, 91.00) | <0.001 |
| CRP (mg/L) | 29.94 (8.40-49.75) | 1.60 (0.97, 2.45) | 33.86 (12.25, 52.72) | <0.001 |
| WBC (*10^9^/L) | 6.11 (4.91-7.75) | 5.35 (4.12, 6.66) | 6.21 (5.03, 7.89) | <0.001 |
| Neut (*10^9^/L) | 3.94 (2.88-5.28) | 3.07 (2.11, 4.27) | 4.04 (3.00, 5.37) | <0.001 |
| Lymph (*10^9^/L) | 1.49 (1.16-1.89) | 1.81±0.65 | 1.46 (1.15, 1.86) | <0.001 |
| Mono (*10^9^/L) | 0.44 (0.34-0.58) | 0.41 (0.31, 0.53) | 0.45 (0.34, 0.59) | 0.024 |
| RBC (*10^12^/L) | 3.86±0.50 | 3.94±0.51 | 3.85±0.50 | 0.084 |
| HGB (g/L) | 110.00 (99.00-122.00) | 115.74±17.40 | 110.00 (99.00, 121.00) | 0.001 |
| PLT (*10^9^/L) | 278.00 (223.00-344.00) | 243.00 (214.50, 288.00) | 282.00 (226.00, 351.00) | <0.001 |
| Albumin (g/L) | 36.65±5.02 | 40.27±4.21 | 36.26±4.94 | <0.001 |
| Globulin (g/L) | 32.30 (28.80-36.20) | 28.98±4.80 | 32.70 (29.15, 36.70) | <0.001 |
| DAS28-ESR | 4.57 (3.96-5.08) | 3.68±0.75 | 4.63 (4.06 5.13) | <0.001 |
| DAS28-CRP | 3.84 (3.28-4.33) | 2.73±0.54 | 3.94 (3.44, 4.40) | <0.001 |

**Note:** We define patients in the queue with CALLY values at the highest decile (>2.16, based on the 90th percentile) as “Highest CALLY Decile”.

**Abbreviations:** *CALLY* C-reactive protein-albumin-lymphocyte index, *MH* medical history, *BMI* body mass index, *csDMARDs* disease-modifying antirheumatic drugs, *b/tsDMARDs* biologic or targeted synthetic DMARDs, *ESR* erythrocyte sedimentation rate, *CRP* high-sensitivity C-reactive protein, *WBC* white blood cell count, *Neut* neutrophil count, *Lymph* lymphocyte count, *Mono* monocyte count, *RBC* red blood cell, *HGB* hemoglobin, *PLT* platelet, *DAS28* 28-joint Disease Activity Score.

**Supplementary Table S2.** Detailed Use of Antirheumatic Drugs in the Study Cohort (N=1,058)

| Medication Category & Specific Drug | Number of Patients | Percentage, % |
| --- | --- | --- |
| Traditional disease modifying antirheumatic drugs (csDMARDs) | 506 | 47.80 |
| Methotrexate | 203 | 19.19 |
| Leflunomide | 205 | 19.38 |
| Hydroxychloroquine | 59 | 5.58 |
| Sulfasalazine | 34 | 3.21 |
| Iguratimod | 144 | 13.61 |
| Patients on ≥1 csDMARD | 374 | 35.30 |
| Patients on ≥2 csDMARDs | 132 | 12.50 |
| Biologic & Targeted Synthetic DMARDs (b/tsDMARDs) | 73 | 6.90 |
| Tumor Necrosis Factor Inhibitors (TNFi) | 40 | 3.78 |
| Etanercept | 19 | 1.80 |
| Adalimumab | 21 | 1.98 |
| Interleukin-6 Receptor Antagonist (Tocilizumab) | 4 | 0.38 |
| JAK Inhibitor (Tofacitinib) | 29 | 2.74 |
| Other Concomitant Medications | - | - |
| Glucocorticoids | 115 | 10.90 |

**Note:** Data are presented as number of patients and column percentage. Percentages may not sum to 100% due to combination therapies.

**Abbreviations:** *JAK* Janus kinase.

**Supplementary Table S3.** Baseline Characteristics by Disease Duration Group

| Characteristics | Total | Early RA (≤2 years) | Established RA (>2 years) | *P* value |
| --- | --- | --- | --- | --- |
| N | 1058 | 345 | 713 |  |
| Sex, % | | | | 0.137 |
| Female | 832 (78.60%) | 262 (75.94%) | 570 (79.94%) |  |
| Male | 226 (21.40%) | 83 (24.06%) | 143 (20.06%) |  |
| Age (year) | 59.00 (51.00-68.00) | 56.00 (46.00, 67.00) | 60.00 (52.00, 68.00) | 0.002 |
| MH (month) | 70.00 (12.00-132.00) | 6.00 (3.00, 12.00) | 98.62 (68.00, 180.00) | ＜0.001 |
| BMI (kg/m^2^) | 23.71 (21.26-26.04) | 24.17±3.39 | 23.69±3.62 | 0.040 |
| Glucocorticoids,% | | | | ＜0.001 |
| No | 943 (89.10%) | 325 (94.20%) | 618 (86.68%) |  |
| Yes | 115 (10.90%) | 20 (5.80%) | 95 (13.32%) |  |
| csDMARDs, % | | | | ＜0.001 |
| No | 552 (52.20%) | 228 (66.09%) | 324 (45.44%) |  |
| Yes | 506 (47.80%) | 117 (33.91%) | 389 (54.56%) |  |
| Type of csDMARDs,% | | | | ＜0.001 |
| 0 | 552 (52.20%) | 228 (66.09%) | 324 (45.44%) |  |
| 1 | 374 (35.30%) | 96 (27.83%) | 278 (38.99%) |  |
| ≥2 | 132 (12.50%) | 21 (6.08%) | 111 (15.57%) |  |
| b/tsDMARDs,% | | | | ＜0.001 |
| No | 985 (93.10%) | 338 (97.97%) | 647 (90.74%) |  |
| Yes | 73 (6.90%) | 7 (2.03%) | 66 (9.26%) |  |
| ESR (mm/H) | 61.00 (37.00-89.00) | 62.40 (36.00, 85.00) | 61.00 (37.00, 90.00) | 0.473 |
| CRP (mg/L) | 29.94 (8.40-49.75) | 28.78 (7.30, 43.51) | 30.93 (8.82, 52.30) | 0.026 |
| WBC (*10^9^/L) | 6.11 (4.91-7.75) | 6.36 (5.22, 7.77) | 6.02 (4.75, 7.74) | 0.015 |
| Neut (*10^9^/L) | 3.94 (2.88-5.28) | 4.19 (3.15, 5.40) | 3.85 (2.78, 5.20) | 0.005 |
| Lymph (*10^9^/L) | 1.49 (1.16-1.89) | 1.54 (1.19, 1.90) | 1.48 (1.14, 1.88) | 0.530 |
| Mono (*10^9^/L) | 0.44 (0.34-0.58) | 0.44 (0.34, 0.57) | 0.44 (0.34, 0.58) | 0.984 |
| RBC (*10^12^/L) | 3.86±0.50 | 3.95±0.48 | 3.82±0.50 | ＜0.001 |
| HGB (g/L) | 110.00 (99.00-122.00) | 113.00 (104.00, 124.50) | 109.00±17.36 | ＜0.001 |
| PLT (*10^9^/L) | 278.00 (223.00-344.00) | 285.00 (231.50, 345.50) | 274.00 (220.00, 344.00) | 0.172 |
| Albumin (g/L) | 36.65±5.02 | 36.90±5.02 | 36.54±5.02 | 0.276 |
| Globulin (g/L) | 32.30 (28.80-36.20) | 32.70 (29.30, 35.90) | 32.10 (28.50, 36.20) | 0.284 |
| DAS28-ESR | 4.57 (3.96-5.08) | 4.65 (4.08, 5.12) | 4.49 (3.93, 5.04) | 0.025 |
| DAS28-CRP | 3.84 (3.28-4.33) | 3.90 (3.36, 4.33) | 3.81 (3.24, 4.33) | 0.169 |
| CALLY | 0.20 (0.10, 0.70) | 0.22 (0.11, 0.86) | 0.20 (0.09, 0.65) | 0.034 |

**Abbreviations:** *CALLY* C-reactive protein-albumin-lymphocyte index, *MH* medical history, *BMI* body mass index, *csDMARDs* disease-modifying antirheumatic drugs, *b/tsDMARDs* biologic or targeted synthetic DMARDs, *ESR* erythrocyte sedimentation rate, *CRP* high-sensitivity C-reactive protein, *WBC* white blood cell count, *Neut* neutrophil count, *Lymph* lymphocyte count, *Mono* monocyte count, *RBC* red blood cell, *HGB* hemoglobin, *PLT* platelet, *DAS28* 28-joint Disease Activity Score.

**Supplementary Table S4.** Univariate Analysis of Factors Associated with DAS28 by Disease Duration

| Characteristics | Early RA (≤2 years) | | Established RA (>2 years) | |
| --- | --- | --- | --- | --- |
|  | DAS28-ESR  β (95% CI) | DAS28-CRP  β (95% CI) | DAS28-ESR  β (95% CI) | DAS28-CRP  β (95% CI) |
| Sex, N (%) | | | | |
| Female | Ref | Ref | Ref | Ref |
| Male | -0.119 (-0.304, 0.067) | **-0.354 (-0.526, -0.183)** | -0.029 (-0.178, 0.121) | **-0.186 (-0.329, -0.044)** |
| Age (year) | 0.002(-0.002, 0.006) | 0.003 (-0.001, 0.007) | -0.003 (-0.007, 0.001) | -0.003 (-0.007, 0.000) |
| MH (month) | **-0.010 (-0.017, -0.003)** | **-0.008 (-0.015, -0.002)** | 0.000 (0.000, 0.001) | 0.000 (0.000, 0.001) |
| BMI (kg/m^2^) | -0.004 (-0.019, 0.011) | -0.008 (-0.023, 0.006) | 0.006 (-0.005, 0.017) | 0.001 (-0.010, 0.013) |
| Glucocorticoids, N (%) | | | | |
| No | Ref | Ref | Ref | Ref |
| Yes | 0.220 (-0.120, 0.559) | 0.194 (0.225, -0.120) | -0.086 (-0.264, 0.093) | -0.064 (-0.235, 0.106) |
| csDMARDs, N (%) | | | | |
| No | Ref | Ref | Ref | Ref |
| Yes | **-0.304 (-0.474, -0.135)** | **-0.290 (-0.446, -0.134)** | **-0.231 (-0.355, -0.108)** | **-0.258 (-0.376, -0.140)** |
| b/tsDMARDs | | | | |
| No | Ref | Ref | Ref | Ref |
| Yes | -0.320 (-0.884, 0.244) | -0.423 (-0.944, 0.097) | **-0.245 (-0.452, -0.038)** | -0.194 (-0.392, 0.004) |
| ESR (mm/H) | **0.016 (0.013, 0.018)** | **0.004 (0.001, 0.002)** | **0.015 (0.013, 0.017)** | **0.005 (0.003, 0.007)** |
| CRP (mg/L) | 0.000 (-0.002, 0.002) | **0.006 (0.004, 0.008)** | 0.001 (-0.001, 0.001) | **0.006 (0.005, 0.008)** |
| WBC (*10^9^/L) | 0.062 (-0.156, 0.280) | 0.018 (-0.192, 0.228) | -0.014 (-0.096, 0.067) | -0.010 (-0.093, 0.072) |
| Neut (*10^9^/L) | -0.033 (-0.251, 0.185) | 0.006 (-0.204, 0.216) | 0.034 (-0.048, 0.117) | 0.027 (-0.056, 0.111) |
| Lymph (*10^9^/L) | 0.001 (-0.249, 0.250) | 0.065 (-0.176, 0.305) | **0.121 (0.011, 0.231)** | **0.125 (0.014, 0.236)** |
| Mono (*10^9^/L) | -0.150 (-0.556, 0.255) | 0.049 (-0.342, 0.440) | -0.100 (-0.364, 0.165) | -0.012 (-0.280, 0.256) |
| RBC (*10^12^/L) | 0.010 (-0.171, 0.192) | 0.085 (-0.090, 0.260) | -0.057 (-0.197, 0.084) | -0.019 (-0.161, 0.123) |
| HGB (g/L) | -0.001 (-0.006, 0.005) | -0.001 (-0.006, 0.004) | 0.002 (-0.003, 0.006) | 0.002 (-0.002, 0.006) |
| PLT (*10^9^/L) | 0.000 (-0.001, 0.000) | 0.000 (-0.001, 0.000) | 0.000 (0.000, 0.001) | 0.000 (0.000, 0.001) |
| Albumin (g/L) | -0.003 (-0.015, 0.010) | -0.006 (-0.018, 0.007) | **-0.016 (-0.025, -0.007)** | **-0.019 (-0.028, -0.010)** |
| Globulin (g/L) | 0.011 (-0.001, 0.024) | 0.011 (-0.001, 0.023) | **0.009 (0.001, 0.018)** | **0.010 (0.001,0.019)** |
| CALLY | **-0.054 (-0.088, -0.021)** | **-0.149 (-0.181, -0.117)** | **-0.064 (-0.096, -0.032)** | **-0.150 (-0.182, -0.118)** |

**Note:** Bold values indicate statistical significance (P < 0.05).

**Abbreviations**: *MH* medical history, *BMI* body mass index, *csDMARDs* disease-modifying antirheumatic drugs, *b/tsDMARDs* biologic or targeted synthetic DMARDs, *ESR* erythrocyte sedimentation rate, *CRP* high-sensitivity C-reactive protein, *WBC* white blood cell count, *Neut* neutrophil count, *Lymph* lymphocyte count, *Mono* monocyte count, *RBC* red blood cell, *HGB* hemoglobin, *PLT* platelet, *CALLY* C-reactive protein-albumin-lymphocyte index, *DAS28* 28-joint Disease Activity Score.

**Supplementary Table S5.** Multivariate Linear Regression by Disease Duration Group

| Exposure | Early RA (≤2 years) | Established RA (>2 years) |
| --- | --- | --- |
| DAS28-ESR | | |
| CALLY (continuous) | | |
| Crude model | -0.054 (-0.088, -0.021) | -0.149 (-0.181, -0.117) |
| Adjust I | -0.182 (-0.224, -0.141) | -0.239 (-0.278, -0.200) |
| Adjust II | -0.141 (-0.182, -0.101) | -0.180 (-0.217, -0.143) |
| CALLY (Tertiles) | | |
| T1 | Ref | Ref |
| T2 | -0.372 (-0.543, -0.202) | -0.379 (-0.501, -0.256) |
| T3 | -0.989 (-1.158, -0.821) | -1.071 (-1.195, -0.948) |
| *P* for trend | ＜0.001 | ＜0.001 |
| DAS28-CRP | | |
| CALLY (continuous) | | |
| Crude model | -0.064 (-0.096, -0.032) | -0.150 (-0.182, -0.118) |
| Adjust I | -0.243 (-0.277, -0.208) | -0.288 (-0.323, -0.253) |
| Adjust II | -0.212 (-0.245, -0.178) | -0.235 (-0.269, -0.202) |
| CALLY (Tertiles) | | |
| T1 | Ref | Ref |
| T2 | -0.368 (-0.500, -0.236) | -0.480 (0.583, -0.376) |
| T3 | -1.234 (-1.364, -1.103) | -1.293 (-1.398, -1.189) |
| *P* for trend | ＜0.001 | ＜0.001 |

Crude model model adjust for: None.

Adjust I model adjust for: sex; age; medical history; BMI.

Adjust II model adjust for: sex; age; medical history; BMI; neutrophil count; monocyte count; red blood cell; hemoglobin; platelet; glucocorticoids; b/tsDMARDs; type of csDMARDs.

**Abbreviations**: *CALLY* C-reactive protein-albumin-lymphocyte index, *ESR* erythrocyte sedimentation rate, *CRP* high-sensitivity C-reactive protein, *DAS28* 28-joint Disease Activity Score, *BMI* body mass index, *csDMARDs* disease-modifying antirheumatic drugs, *b/tsDMARDs* biologic or targeted synthetic DMARDs.

**Supplementary Table S6.** Two-Piecewise Linear Regression by Disease Duration

|  | Early RA (≤2 years) | Established RA (>2 years) |
| --- | --- | --- |
| DAS28-ESR | | |
| Inflection points of CALLY | 0.407 | 0.551 |
| <Inflection point | -2.475 (-3.407, -1.543) | -1.991 (-2.422, -1.560) |
| >Inflection point | -0.081 (-0.130, -0.033) | -0.090 (-0.135, -0.044) |
| *P* for log likelihood ratio test | ＜0.001 | ＜0.001 |
| DAS28-CRP | | |
| Inflection points of CALLY | 0.533 | 0.552 |
| <Inflection point | -2.201 (-2.679, -1.723) | -2.413 (-2.763, -2.063) |
| >Inflection point | -0.117 (-0.154, -0.079) | -0.104 (-0.141, -0.066) |
| *P* for log likelihood ratio test | ＜0.001 | ＜0.001 |

**Note:** The models were adjusted for sex; age; medical history; BMI; neutrophil count; monocyte count; red blood cell; hemoglobin; platelet; glucocorticoids; b/tsDMARDs; type of csDMARDs.

**Abbreviations:** *CALLY* C-reactive protein-albumin-lymphocyte index, *ESR* erythrocyte sedimentation rate, *CRP* high-sensitivity C-reactive protein, *DAS28* 28-joint Disease Activity Score, *BMI* body mass index, *csDMARDs* disease-modifying antirheumatic drugs, *b/tsDMARDs* biologic or targeted synthetic DMARDs.
